# Improving Small-Molecule Force Field Parameters in Ligand Binding Studies

**DOI:** 10.3389/fmolb.2021.760283

**Published:** 2021-12-13

**Authors:** Stefano Raniolo, Vittorio Limongelli

**Affiliations:** ^1^ Faculty of Biomedical Sciences, Euler Institute, Università della Svizzera italiana (USI), Lugano, Switzerland; ^2^ Department of Pharmacy, University of Naples “Federico II”, Naples, Italy

**Keywords:** benzamidine, trypsin, CGenFF, GAFF, QM calculations, free-energy calculations, force fields

## Abstract

Small molecules are major players of many chemical processes in diverse fields, from material science to biology. They are made by a combination of carbon and heteroatoms typically organized in system-specific structures of different complexity. This peculiarity hampers the application of standard force field parameters and their *in silico* study by means of atomistic simulations. Here, we combine quantum-mechanics and atomistic free-energy calculations to achieve an improved parametrization of the ligand torsion angles with respect to the state-of-the-art force fields in the paradigmatic molecular binding system benzamidine/trypsin. Funnel-Metadynamics calculations with the new parameters greatly reproduced the high-resolution crystallographic ligand binding mode and allowed a more accurate description of the binding mechanism, when the ligand might assume specific conformations to cross energy barriers. Our study impacts on future drug design investigations considering that the vast majority of marketed drugs are small-molecules.

## Introduction

Small molecules are organic compounds of relatively low molecular weight which are responsible of specific chemical reactions. Their range of application is broad and spans from material science to pharmacology where they historically represent the end product of drug discovery. Understanding the mechanism of action of a drug by elucidating the binding interaction with its molecular target is fundamental to speed up the drug discovery pipeline, however it might be a long and daunting process, generally requiring extensive and costly experiments (Lindsay, 2003; Tian et al., 2013; Tulloch et al., 2018). In addition, this effort is seldom rewarded since most drug candidates end up in failure due to poor pharmacokinetics properties or the interaction with off-targets (Lindsay, 2003; Waring et al., 2015; Mao et al., 2016). Nonetheless, few of them are successful and in even fewer cases the ligand/protein bound poses are experimentally resolved by X-ray crystallography, providing important insights at atomistic level on the possible interaction mechanism and the binding site in the target molecule. Such information is invaluable for a *de novo* drug design campaign on that target or to modify the ligand structure in order to improve its activity and toxicity, but it is available merely for a selection of drugs. Therefore, the development of reliable and relatively fast computational techniques capable of inferring mechanisms of binding in ligand/protein complexes has taken the scene.

In this scenario, molecular dynamics (MD) simulations and the related enhanced sampling techniques are gaining ground in the years, being able to reproduce crystallographic binding poses and compute absolute binding free energies in agreement with experimental data (Limongelli et al., 2013; Troussicot et al., 2015; Broomhead and Soliman, 2016; Yuan et al., 2018; Limongelli, 2020; Raniolo and Limongelli, 2020; Souza et al., 2020; Monticelli et al., 2021). The possibility to extrapolate atomistic details at relatively low cost makes these computational approaches invaluable tools both to assist drug design and to shed light on important features of biologically relevant processes. In particular, the development of enhanced sampling techniques and the growing computing power are allowing to alleviate the sampling issue with simulation times even closer to the real timescales of ligand/protein binding. However, the precision and accuracy of the physics applied in MD simulations are highly dependent on the reliability of the chosen force fields (FF), which implement properties of atoms - or beads in the case of coarse-grained (CG) MD - that compose the system under investigation. There are plenty of molecular FFs available and all of them are based on data extracted from experimental assays or quantum mechanical (QM) calculations (Mackerell Jr, 2004). Although they approximate the known properties of molecules, FFs yet allow reproducing complicated systems, such as ligand/protein complexes, with a fair accuracy. On the other hand, standalone QM calculations might ideally provide more accurate results, but they are unfeasible due to the enormous amount of atoms to consider and the sheer complexity of the calculation.

The first biologically-related FFs considered mainly proteins, nucleotides, lipids, and carbohydrates, while small drug-like compounds had been neglected in the first stage of the FFs’ life. Eventually, we saw in the last decades the birth of a number of small-molecule FFs, such as the Generalized Amber Force Field (GAFF and its more recent version GAFF2), the CHARMM General Force Field (CGenFF), and OPLS3 among the most famous (Wang et al., 2004; Vanommeslaeghe et al., 2010; Harder et al., 2016). Lately, an extended version of the OPLS FF has been published (i.e., OPLS3e and OPLS4), featuring improved torsional angle description and a wider coverage of the chemical space (Roos et al., 2019; Lu et al., 2021). The main difficulty in developing such FFs is the huge variety of scaffolds and functional groups that a ligand can possess, and taking into consideration all of them requires an astonishing amount of experimental information and/or QM calculations. Therefore, most of the FFs only consider a smaller sample of compounds, trying to extrapolate general rules for all the possible cases. This operation might lead to severe approximations in the generation of the ligand parameters and a manual correction from the investigator is often required. This is especially true in torsion angle parameterization of ligands with *π* electron conjugated systems, where quantum (ligand-specific) effects are particularly important in defining the correct molecular conformation. In this article, we showcase the effect of these problems for benzamidine, a small molecule binding to the protein trypsin, composed of an amidine group conjugated to a benzene ring and used as prototypical model in drug/protein binding studies. Our results show important changes in sampling behaviour of the small molecule depending on different parametrizations and their consequences when free energy calculations are performed to study the binding mechanism with its molecular target.

## Materials and Methods

In this section we describe the process to obtain the benzamidine parameters using the most widely used and freely available FFs for ligands (GAFF, GAFF2, and CGenFF); the simulations employed to obtain the benzamidine conformational potential energy; and the binding free-energy calculations between benzamidine and trypsin.

In order to simulate the molecular binding process, it is necessary to set the parameters to describe the properties of both benzamidine and trypsin. Regarding the latter, there are several optimised FFs for proteins, which have reached a satisfactory level of accuracy. On the contrary, small molecules are made by different combinations of diverse atoms that render unfeasible to catalogue ligands as done for the amino acids in a protein. Therefore, for benzamidine new parameters have to be created either automatically (e.g., from software that try to infer molecular properties) or manually. Among the available FFs for trypsin, we opted for Amber14SB, which represents an improvement with respect to the previous Amber99SB version (Maier et al., 2015). Regarding benzamidine, new parameters compatible with Amber14SB had to be generated. This is possible by employing the Amber “generalised” parameters collected inside the GAFF library, which contains several atom types, bonded and non-bonded parameters to describe the sampling behaviour of organic molecules. In 2016, a new version of this library (i.e., GAFF2) was released to account for improved torsional characterisation and molecular properties, such as intermolecular energy, liquid density, heat of vaporisation, and hydration free energy.

The parametrisation of trypsin using Amber14SB was quite straightforward, contrarily to benzamidine that requested a “construction protocol”, which was inspired by the GAFF reference paper and that will be described in the following section (Wang et al., 2004).

### Ligand Parametrization

The first step is to perform a geometry optimization of the benzamidine structure using the QM software Gaussian09 (Frisch et al., 2009). This step was divided in two different optimisation steps with increasing basis set complexity (3-21G and 6-31G*, respectively), where a Hartree-Fock calculation was requested, followed by a Möller-Plesset correlation energy correction truncated at the second order (MP2) (Møller and Plesset, 1934; Gordon et al., 1982; Petersson et al., 1988). Total charge for the system was set to +1, since in solution at physiological pH the amidine group is protonated (*pk*_*a*_ = 11.6) (Lam et al., 2003). Once obtained the optimised structure, we performed a population analysis with Hartree-Fock to produce charges considering the electrostatic potential at points following the Merz-Singh-Kollman scheme (Besler et al., 1990). The output file was post-processed by the Restrained Electrostatic Potential (RESP) method to obtain the partial charges per atom (Bayly et al., 1993). Restraints in the charge allocation were applied to account for benzamidine symmetry.

The atomic charges thus obtained were used to create the topology of benzamidine in the case of GAFF and GAFF2, whereas CGenFF already has tabulated charges for the molecule so we applied them for internal consistency with the rest of the parameters. Notably, the CGenFF charges differ from those obtained through RESP, especially for the amidine group, as listed in Table 1.

**TABLE 1:** Table reporting the atomic charges for benzamidine in CGenFF and RESP.

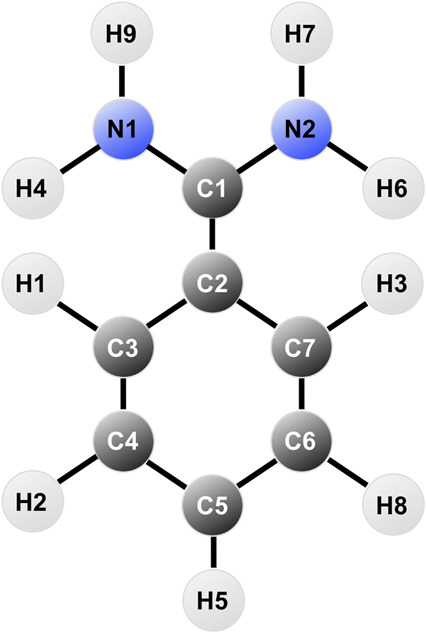

Atom	CGenFF	RESP
N1	−0.600	−0.901011
H4	0.320	0.447034
H9	0.320	0.474397
C1	0.730	0.798507
N2	−0.600	−0.901011
H6	0.320	0.447034
H7	0.320	0.474397
C2	0.190	−0.102912
C3	−0.115	−0.121702
H1	0.115	0.157313
C4	−0.115	−0.136868
H2	0.115	0.173449
C5	−0.115	−0.045171
H5	0.115	0.164353
C6	−0.115	−0.136868
H8	0.115	0.173449
C7	−0.115	−0.121702
H3	0.115	0.157313

The bonded parameters for GAFF and GAFF2 were created using the Antechamber package of Amber14 (Wang et al., 2004). A new *ad hoc* topology was also produced by taking as a template the parameters from GAFF and modifying the dihedral angle along the bond between amidine and benzene. The role of this peculiar dihedral angle will be better highlighted in the “*Results and Discussion*” section. The *ad hoc* parameters were obtained by means of a “Scan” calculation using the settings of the second geometry optimisation process. Such procedure involves a number of energy minimisation calculations in which the dihedral angle of interest is restrained in a specific conformation and relaxing the rest of the molecule. Once obtained the energy profile for the dihedral angle under investigation through QM calculations, we resolved the parameters that reproduced the QM potential energy function using the Amber dihedral formula *E*_*dih*_ = *k* (1 + cos (*nϕ* − *ψ*)), with k being the spring constant for the dihedral angle, n the period, *ϕ* the angle, and *ψ* the phase. The function that best reproduced the QM behaviour was computed with an in-house genetic algorithm and it is a combination of two torsional angles that has the equation *E*_*dih*_ = 2.4 (1 + cos (2*ϕ* − *π*)) + 1.0 (1 + cos (4*ϕ*)). A similar protocol is reported in the GAFF reference manuscript and has already been employed in literature for other compounds (Pophristic et al., 2006). The new parameters, together with adjustments to angle values taken from the QM geometry optimisation, replaced the original GAFF values and represent our *ad hoc* topology.

### Ligand Conformational Analysis

The simulations with the all-atom FFs were performed using well-tempered metadynamics (MetaD) (Barducci et al., 2008), using the torsional angle connecting the amidine and the phenyl group as collective variable (i.e., the degree of freedom that we are going to sample), and compared the obtained energy profiles with that from QM calculations. The MetaD calculations were run using Amber14 patched with Plumed-2.3.3 (Tribello et al., 2014), setting a low value as height of the metadynamics Gaussian functions (i.e., 0.1 kJ/mol for CGenFF and the *ad hoc* topology and 0.05 kJ/mol for GAFF and GAFF2), a sigma of 0.05 radians, a biasfactor of 15, and a deposition rate of 1,000 steps. All simulations were carried out in vacuum and converged within 100 ns (see Supplementary Figure S1 in the “Supplementary Material” for convergence plots). Post-processing analysis was performed with Plumed-2.3.3 and Visual Molecular Dynamics (VMD) (Humphrey et al., 1996; Tribello et al., 2014).

### MD Calculations on the Benzamidine/Trypsin Complex

Classical MD simulations on the benzamidine/trypsin complex in water were performed with the Amber14 software to assess the stability of benzamidine in the binding pocket employing GAFF and *ad hoc* ligand parametrization (Case et al., 2014; Abraham et al., 2015). We produced 500 ns of simulation of benzamidine in the bound pose with the Amber14SB FF and water model TIP3P.

### Binding Free-Energy Calculations on the Benzamidine/Trypsin Complex

Funnel-Metadynamics simulations were used to obtain the binding free-energy surface of the benzamidine/trypsin complex (Limongelli et al., 2013). The GAFF free energy data were taken from our previous work (Limongelli et al., 2013), while new calculations were performed to build the *ad hoc* binding free energy surface. In particular, the protocol we have recently reported in Nature Protocols was employed (Raniolo and Limongelli, 2020). More details on the Funnel-Metadynamics calculations can be found in the “Supplementary Material” and the reference manuscript (Raniolo and Limongelli, 2020). As reported in Limongelli *et al.* (Limongelli et al., 2013), the estimate of the absolute ligand/protein binding free-energy ( $\Delta G_{b}^{0}$ ) was obtained using the following formula:

$$K_{b}=\pi R_{cyl}^{2}\int_{site}e^{-\beta \left[W\left(z\right)-W_{ref}\right]}\;dz$$ (1)

$$\Delta G_{b}^{0}=-k_{b}Tln\left(K_{b}C^{0}\right)$$ (2)

where K_*b*_ is the binding constant, R_*cyl*_ is the radius of the cylinder section of the FM potential, *β* is the inverse of the Boltzmann constant (k_*b*_) multiplied by temperature (T), W(z) is the potential of mean force (PMF) at given value z for the projection of the ligand over the axis of the funnel potential, W_*ref*_ is a reference value of the PMF for the unbound state, and C^0^ is the standard concentration.

## Results and Discussion

**FIGURE 1:**
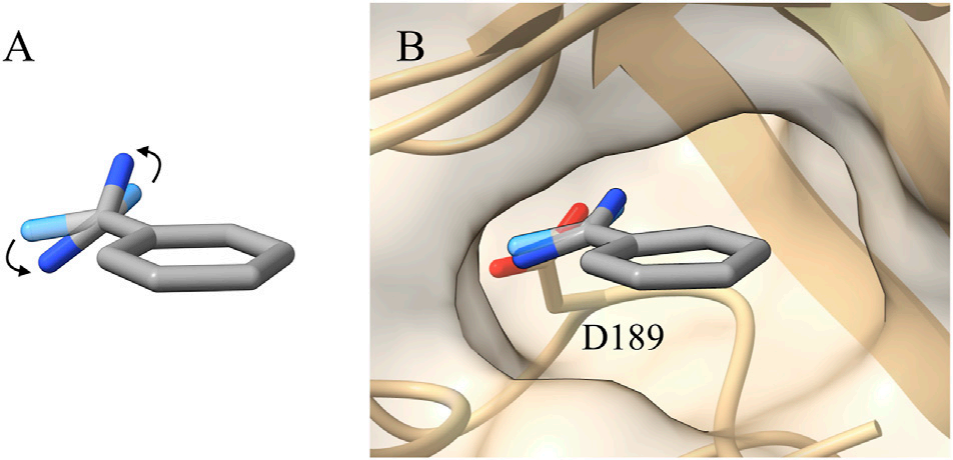
**(A)** Representation of the benzamidine conformation with the tilted amidine group (conformation with the amidine group in planar conformation shown as transparency). **(B)** Benzamidine with the tilted amidine group as resolved in the 0.75 Å high resolution X-ray structure (PDBID 4I8H). In such a conformation, the amidine group is perfectly aligned with the interacting ASP189 of trypsin (Liebschner et al., 2013).

Benzamidine is a small molecule acting as an inhibitor of trypsin and it interacts mainly through an amidine group contacting an aspartate in the binding pocket of the protein (ASP189) (Figure 1).

This complex is widely used as a benchmark system for newly developed computational techniques aimed at disclosing ligand/protein binding modes and calculate binding free energies. The results obtained with a new technique can be indeed compared with those achieved with already established methods and with experimental values obtained through isothermal titration calorimetry (ITC) (Katz et al., 2001; Talhout and Engberts, 2001; Doudou et al., 2009; Buch et al., 2011; Söderhjelm et al., 2012; Limongelli et al., 2013; Takahashi et al., 2014).

While setting up the system, we generated the topology for both benzamidine and trypsin, as reported in the “*Materials and Methods*” section. During the geometrical optimization of benzamidine with Gaussian09, a rotation of the amidine group with respect to the benzene ring was observed, ending up in a *π*/4 radians tilt between the two groups (Figure 1A). The rotation disaligned the two groups, *de facto* partially disrupting the conjugation between them. This behaviour is likely due to the steric clash formed by the hydrogen atoms of the amidine group and those of the benzene ring. In the attempt to resolve the steric hindrance, the amidine group tilts, resulting in the conformation obtained by Gaussian09 (Figure 1A).

It is worth noting that this system has been widely investigated in the past and in several works a planar benzamidine—with the amidine group not tilted - was reported in the benzamidine-trypsin complex (Doudou et al., 2009; Buch et al., 2011; Söderhjelm et al., 2012; Limongelli et al., 2013; Takahashi et al., 2014; Tiwary et al., 2015). However, there are cases in which a thorough analysis has been carried out, obtaining benzamidine geometries similar to the one defined by our calculation (Li et al., 2009).

In order to gain more insights, we compared the Gaussian09 optimised structure with several crystals deposited in the Protein Databank (PDB) and we found a more recent high-resolution structure (0.75 Å, PDBID 4I8H) where the benzamidine with the tilted amidine group was crystallized inside the binding pocket of trypsin (Figure 1B) (Liebschner et al., 2013).

**FIGURE 2:**
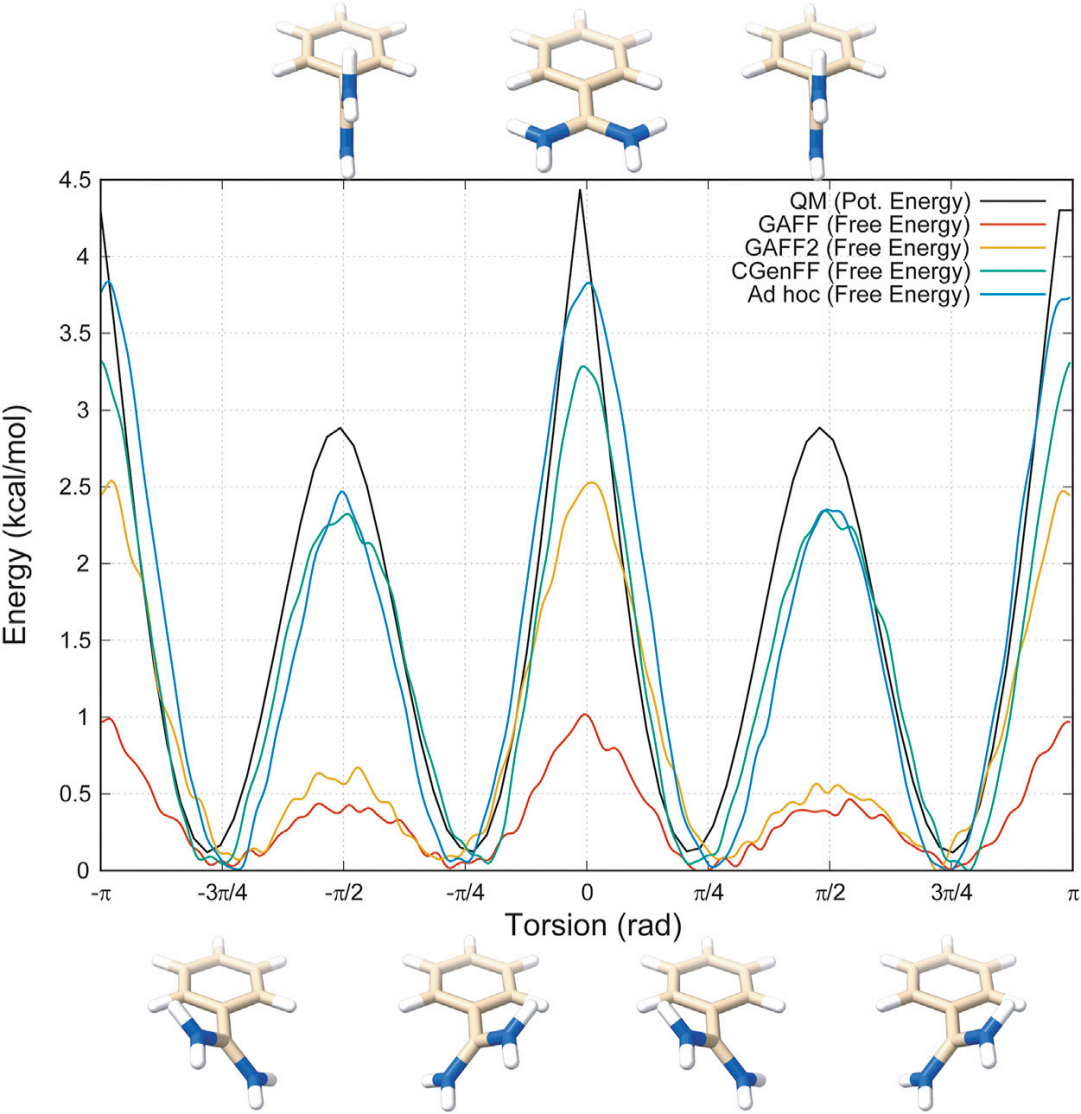
Energy profiles of the dihedral angle between the benzene and the amidine group. In black is represented the one obtained with Gaussian09, in red GAFF, in orange GAFF2, in green CGenFF, and in blue the *ad hoc* parametrization purposely developed for this study.

Then, we decided to investigate more deeply the rotation around the torsional angle responsible for this behaviour and we performed a thorough analysis of the dihedral angle with Gaussian09 (see “*Materials and Methods*” for further details). The results clearly show that the fully planar conformation is highly disfavoured with an energy barrier of around 4.5 kcal/mol, whereas the minima reside at *π*/4 + *nπ*/2 angles, with n being an integer number that takes into account the periodicity of the angle (Figure 2, black line).

This finding contrasts with some of the results previously reported in literature, therefore we proceeded with comparing the conformational energy profile of benzamidine obtained with QM calculations with those computed using the available small-molecule libraries (Doudou et al., 2009; Buch et al., 2011; Söderhjelm et al., 2012; Limongelli et al., 2013; Takahashi et al., 2014; Tiwary et al., 2015). In particular, the topology file of the ligand, necessary to perform the conformational analysis simulations, can be generated using position and point charges of the ligand’s atoms together with the bonded and non-bonded parameters from the FF’s small-molecule library files (i.e., GAFF or GAFF2 using the AmberTools module in the case of the Amber FF). The parameters are assigned based on identity or similarity of the atoms present in the molecule with the atom types reported in the FF library. This procedure might lead to major approximations in the calculation if the ligand’s atoms are not very similar to those already parametrised in the library. Thus, we created the benzamidine topology for a selection of widely used, open-source FFs for small molecules to assess the goodness of the parameters, especially in reproducing the correct conformational behaviour of the dihedral angle connecting the amidine group and the phenyl ring. In particular, we investigated topologies created from the two versions of the Amber GAFF library (GAFF and GAFF2) and the equivalent for the CHARMM-36 FF CGenFF, which includes specific parameters for benzamidine (BAMI). In addition, we created a brand new *ad hoc* topology by modifying the ligand parameters from GAFF in order to replicate as close as possible the torsion potential computed through QM calculations (see the “Materials and Methods” section). Finally, for each topology we performed all-atom metadynamics calculations using as single collective variable (CV) the dihedral angle connecting the amidine group and the benzene ring (see Supplementary Figure S1 in the “Supplementary Materials”).

The obtained conformational energy profiles of benzamidine are compared with that from the QM calculations in Figure 2.

Interestingly, the best energy profile is achieved by means of the *ad hoc* topology with a difference in the estimate of the energy barriers lower than 0.5 kcal/mol with respect to QM (Figure 2, blue line). Conversely, all the Amber and CHARMM FFs underestimate the energy barriers. In particular, GAFF delivers the worst profile showing energy barriers of 1 and 0.5 kcal/mol at the planar and perpendicular conformations, respectively. By using GAFF2, the situation slightly improves with an energy barrier at the planar conformation around 2.5 kcal/mol, closer to 4.5 kcal/mol measured at QM level. On the other hand, the barrier at the perpendicular conformation at ± *π*/2 remains largely underestimated compared to QM. In this scenario, CGenFF performs much better, providing around 3.0 and 2.5 kcal/mol for the barriers at the planar and perpendicular conformation, respectively. On the other hand, the atomic partial charges computed for benzamidine by CGenFF are quite different from the one calculated by QM (see “*Materials and Methods*” for details). This is an important aspect to consider, especially in processes like the benzamidine interaction with trypsin, where the binding is governed mainly by electrostatic contributions between the amidine group of the ligand and the carboxyl group of ASP189. In summary, all the FFs correctly identify the conformation with the tilted amidine group as the lowest energy state, however the Amber FFs severely underestimate the energy barriers that separate the different conformations assumed by benzamidine (Figure 2).

Prompted by these results, we decided to investigate the effect of the different ligand parametrization in its binding interaction to trypsin. To this end, we performed plain all-atom MD calculations on two benzamidine/trypsin systems:

1. using GAFF (replicating the same conditions used in our previous work (Limongelli et al., 2013));
2. using the *ad hoc* topology.

**FIGURE 3:**
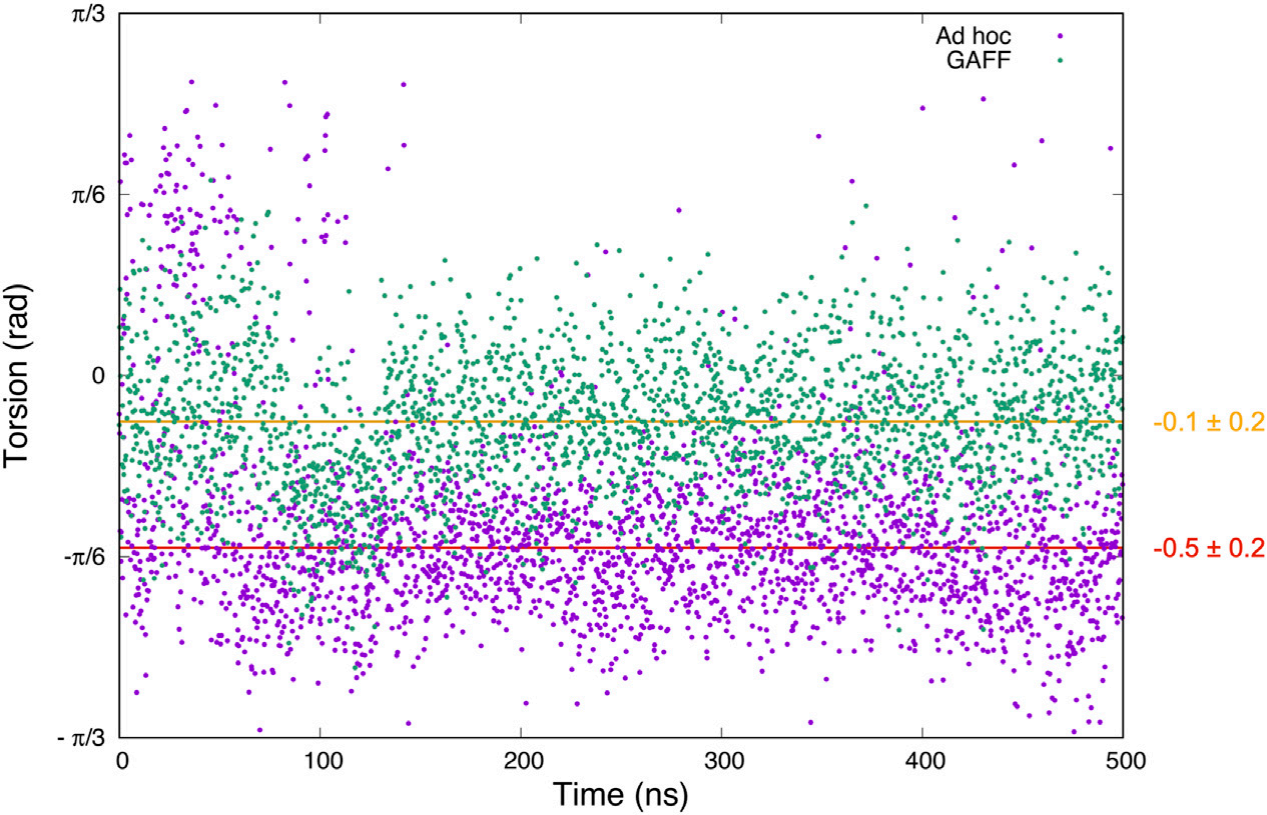
Graph showing the values of the dihedral angle connecting the amidine group and the benzene ring of benzamidine observed along 500 ns of plain MD simulations. In green is the result for the GAFF parameters, while in violet is the one obtained with our *ad hoc* topology. The torsion average values and standard deviation using GAFF and *ad hoc* parameters are shown in orange and red, respectively.

Comparing these simulations, we expected to observe a higher conformational freedom for benzamidine using GAFF, considering the lower energy barriers. The prediction was indeed confirmed (Figure 3).

**FIGURE 4:**
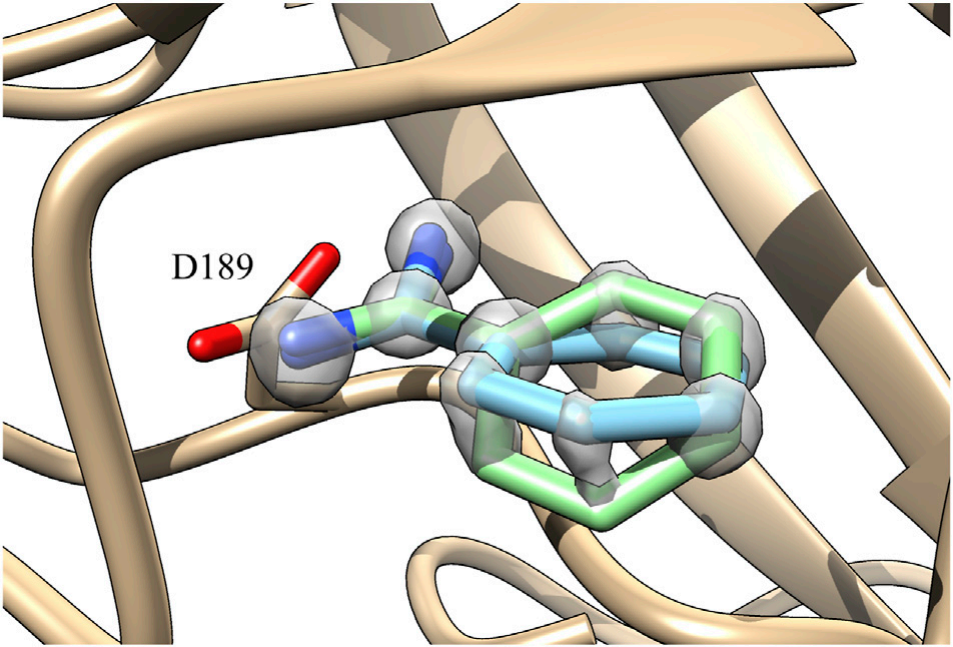
Superimposition with the X-ray electronic density map of the two benzamidine conformations obtained with the *ad hoc* parametrization having the dihedral angle at 0.5 and −0.5 radians shown in green and cyan, respectively. The density map of benzamidine has been represented at a contour level of 2.6 Å and transparency 0.5. The density map of the protein has been undisplayed for clarity reason (Liebschner et al., 2013).

In the case of GAFF, benzamidine’s dihedral fluctuates around the value −0.13 radians (Figure 3, orange line) and it frequently populates the planar conformation (0 radian), whereas the latter is rarely visited using our *ad hoc* parametrization, where the dihedral value fluctuates around −0.5 radians (Figure 3, red line). In particular, in the latter case visiting conformations close to the barrier at 0 radian is energetically disfavoured, while the ligand assumes a more stable conformation with the tilted amidine group in which the nitrogen atoms of benzamidine are perfectly aligned with the oxygens of the carboxylic group of ASP189 in trypsin (Figure 1B). We extracted the structures of the benzamidine/trypsin complex representing the most populated conformational clusters visited during the *ad hoc* simulation at which the studied dihedral angle assumes around ±0.5 radians and we fit them in the electronic density map obtained through high-resolution X-ray experiments by Liebschner et al. (2013). Notably, the two in-silico conformations perfectly fit the map evidencing that our *ad hoc* parametrization allows reproducing the experimental ligand binding mode at a very high atomistic resolution of 0.75 Å (Figure 4).

**FIGURE 5:**
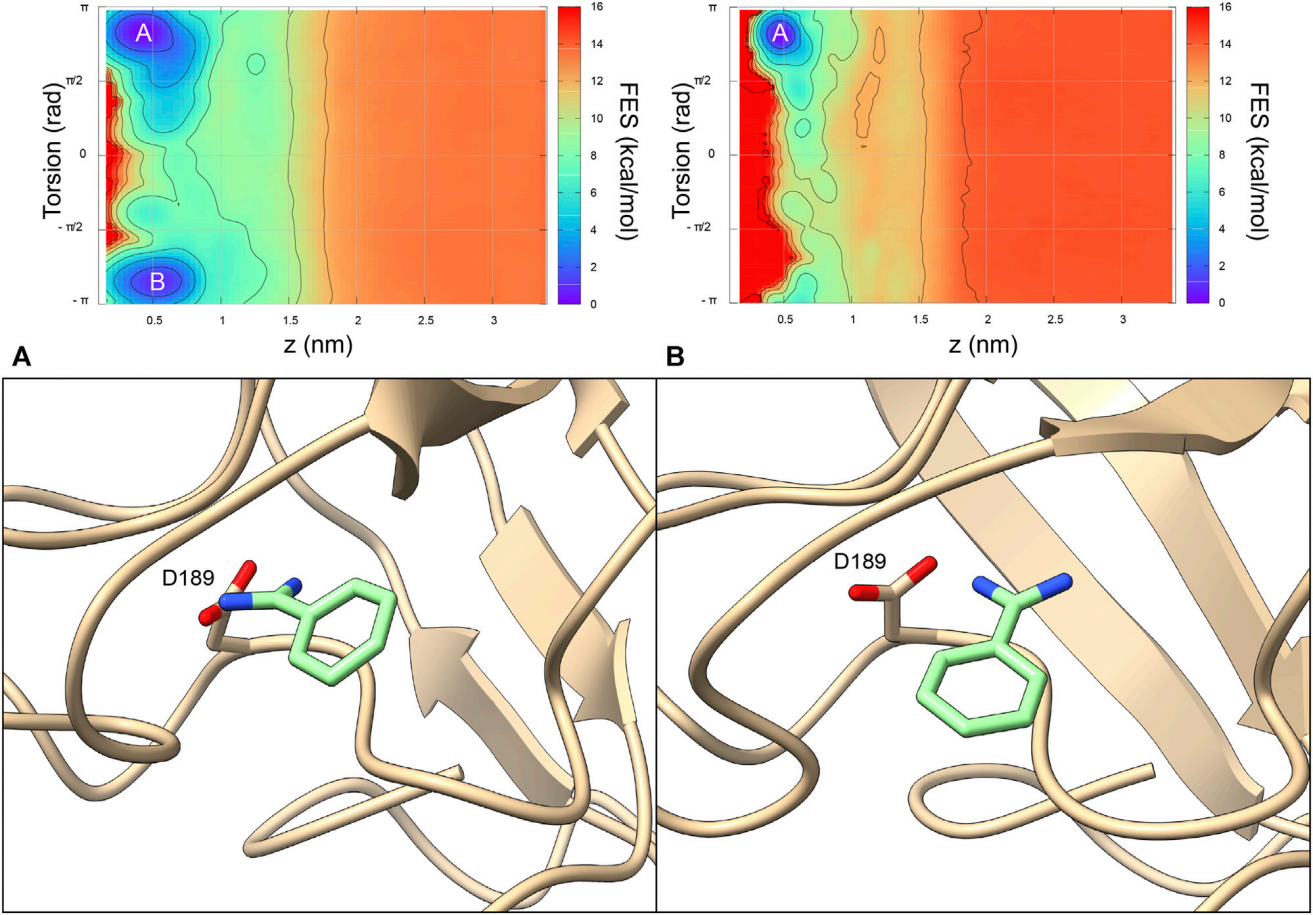
The 2D benzamidine/trypsin binding free-energy surfaces obtained using the benzamidine GAFF topology (Limongelli et al., 2013) **(*Left*)** and the benzamidine *ad hoc* topology **(*Right*)**. The *x* axis represents the distance between the ligand and the protein, while the *y* axis is the orientation of the ligand relative to the protein. At variance with the GAFF simulations, using the *ad hoc* topology the state **(B)** does not represent an energy minimum. Representatives of the clusters of state **(A, B)** are offered to better understand the difference binding mode of benzamidine.

In order to investigate how the different benzamidine parameters affect the ligand binding mechanism to trypsin - intended as the physical pathway followed by the ligand to reach the binding site from its fully solvated state - we performed binding free-energy calculations using Funnel-Metadynamics (Limongelli et al., 2013; Raniolo and Limongelli, 2020) and the two different parametrizations (i.e., using GAFF and the *ad hoc* topology). Details on the procedure are reported in the “*Materials and Methods*” section, while we refer the reader to our recent protocol manuscript for a more extensive description of the methodology, where the ad hoc topology has been employed (Raniolo and Limongelli, 2020). It is worth mentioning that we focused on the comparison with GAFF since the latter was employed in our previous work on the same system using the same charge protocol (Limongelli et al., 2013), thus making the comparison straightforward. In Figure 5, it is possible to see the free-energy landscapes computed in the two cases.

In detail, the absolute binding free-energy value remains almost the same (i.e., −8.5 and −8.2 kcal/mol for the GAFF and *ad hoc* simulation, respectively), falling in the range from −9.0 to −5.5 kcal/mol obtained in previous theoretical and experimental studies (Katz et al., 2001; Talhout and Engberts, 2001; Doudou et al., 2009; Buch et al., 2011; Söderhjelm et al., 2012; Limongelli et al., 2013; Takahashi et al., 2014; Tiwary et al., 2015). However, the number of free-energy minima changes with two minima for GAFF and only one for the *ad hoc* system (Figure 5). In particular, the second lowest energy minimum of the GAFF system (minimum B in the left graph in Figure 5) is characterized by a completely planar conformation assumed by benzamidine. Such state does not represent a low energy minimum in the *ad hoc* system and this is likely due to the higher energy barrier corresponding to the planar ligand conformation. Instead, the crystallographic binding mode was extensively explored and represents the absolute minimum in both cases (letter A in Figure 5). Furthermore, when the ligand is bound, its torsional freedom is much reduced with the *ad hoc* topology, exploring much of the time the crystallographic binding mode (further information in the Supplementary Data and Supplementary Figure S2), which results in a narrower minimum A.

**FIGURE 6:**
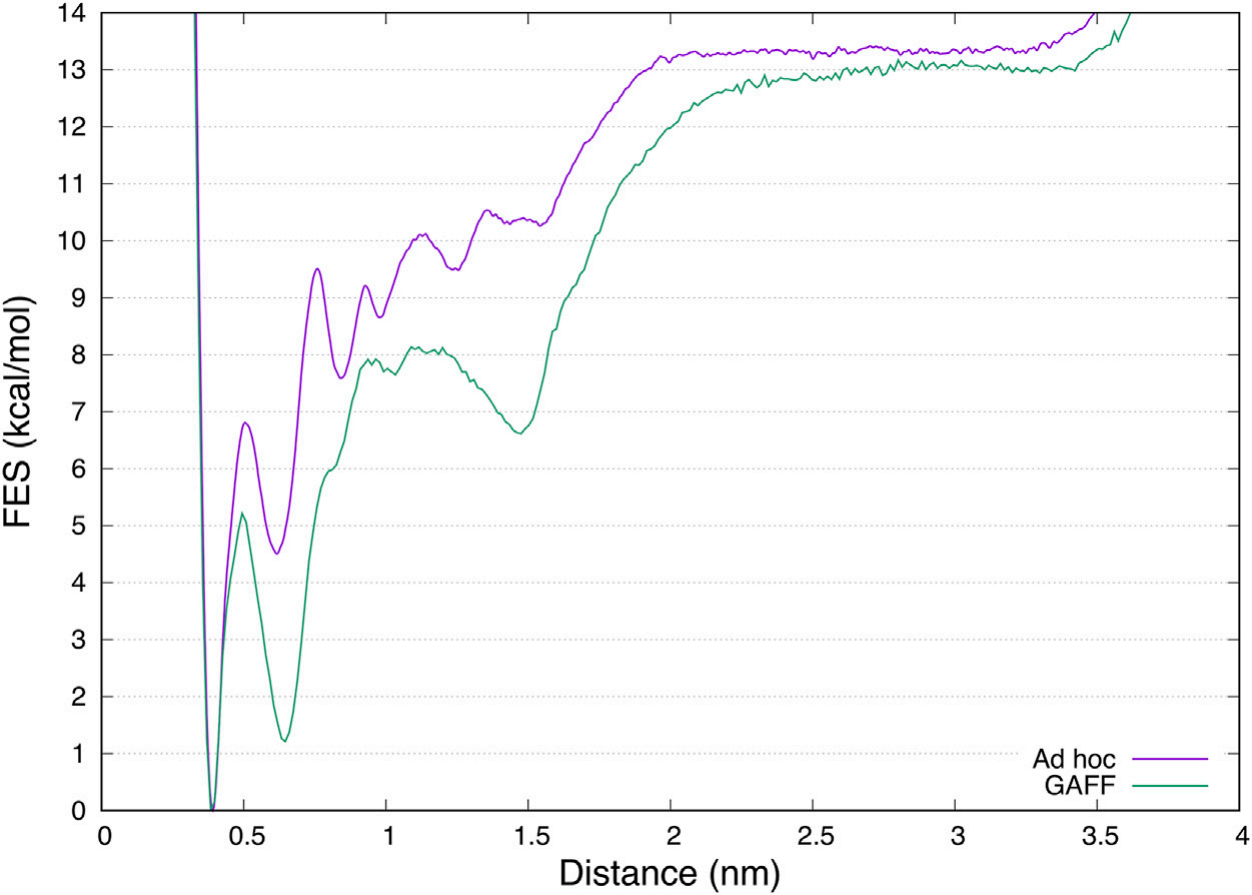
Superimposition of the mono-dimensional free-energy profiles as a function of the distance between benzamidine and trypsin obtained from FM simulations. While the absolute minimum and the unbound region are almost overlapping, the in between region representing the ligand binding mechanism is different, characterized by more barriers at higher energy in the case of the *ad hoc* calculations.

Finally, a different free energy profile for the ligand binding pathway was obtained in GAFF and *ad hoc* system. In particular, in the latter a larger number of states at higher energy values were found (Figure 6), indicating that the ligand has to cross multiple high energy barriers during the binding to pass from one state to another and eventually reach the binding site. This results in longer times of binding and unbinding in the *ad hoc* system if compared with the GAFF one. Such an aspect should be carefully considered if the aim of the investigation is to disclose by means of simulations states determining ligand binding kinetics, and compute association and dissociation rates.

## Conclusion

In the present work, we have carried out a thorough study on ligand parametrization in the prototypical case of the benzamidine/trypsin binding complex. We have demonstrated that having accurate ligand parameters is fundamental to simulate properly the ligand conformational freedom and more importantly its binding mechanism to the molecular target. In fact, during the physical approach of a drug to its molecular target, the ligand might assume specific conformations corresponding to certain values of ligand torsion angles - that are necessary to cross energy barrier and eventually reach the binding site. We have showed how the optimization of a single torsional angle potential deeply affects the free-energy landscape of the binding process, and in turn the characterization of both the thermodynamically and kinetically-relevant states which allow disclosing the ligand binding mode and the state determining the ligand binding rate, respectively. This process is fundamental to achieve an accurate estimate of the ligand binding constant K_*b*_ and ligand binding kinetics rate k_*on*_ and k_*off*_, which are useful to develop more potent ligands. It is worth noting that similar conclusions were achieved by Pophristic et al. and Li et al. who showed that an improved description of the ligand binding mode for arylamide and benzamidinium-like compounds could be obtained through a more accurate force field parametrization (Pophristic et al., 2006; Li et al., 2009). The evidences of our study are expected to impact on future drug design investigations, especially considering that the majority of the marketed drugs has much more complex structures than benzamidine, endowed with *π* electron conjugated systems, which might require a dedicated attention by the investigator and an *ad hoc* parametrization.

## Data Availability

The data generated in this work can be found at Google drive. They are organized in folders thematically named as follows: •QM - output files of the quantum-mechanical calculations employed for geometrical optimization, charge calculation and scanning procedures (input of each job is reported as header of each file); •RESP - input and output files of the calculations of atomistic punctual charges that are subsequently added to the Amber topology file; •simulations - inputs and trajectory files useful to launch and reproduce the simulations results reported in the dihedral_analysis, dihedral_sampling, and distance_analysis folders; •topologies - topology files of all the employed force fields. All QM calculations were performed with Gaussian 09 (Frisch et al., 2009). Gromacs-5.1.4, Gromacs-2016.5 and Amber14 were used as MD engines for the vacuum simulations to study the dihedral angle with different topologies and to analyse the sampling in the presence of the complex (Case et al., 2014; Abraham et al., 2015). All the metadynamics simulations were performed using Plumed 2.3.3 (Tribello et al., 2014). The results displayed in Figure 5 and 6 are from simulations on the benzamidine-trypsin complex reported in our previous works. We report the employed methodology in chapter 1 (“Supplementary Material”) of the Supplementary Material, however more details on data and software employed can be found in Limongelli et al. (2013) and Raniolo and Limongelli (2020).
