# Supplementary material for: Improving Small-Molecule Force Field Parameters in Ligand Binding Studies
Source: Front Mol Biosci. 2021 Dec 13;8:760283. doi: 10.3389/fmolb.2021.760283 (PMC8711133; doi:10.3389/fmolb.2021.760283)
Supplement: Supplementary file 1 [file DataSheet1.pdf]

Supplementary Material

1 SUPPLEMENTARY DATA

The conformational analysis of benzamidine was performed using all-atom FFs and metadynamics calculations. The convergence of the free energy calculation was evaluated through the calculation of the free energy as a function of the simulation time and by monitoring the exploration of the CV space along the simulations (Figure S1).

Funnel Metadynamics simulations were carried out using Gromacs-5.1.4 patched by Plumed-2.3.3 and making use of the new code (Raniolo and Limongelli, 2020). We applied the same simulation setting used in our previous work Limongelli et al. where the GAFF topology was used (Limongelli et al., 2013). In particular, we employed the same distance CV - distance between the Cγ of aspartate 189 and the carbon of the amidine group of benzamidine - using 2 kJ/mol as metadynamics Gaussian height, 0.01 nm as sigma, biasfactor of 20, deposition rate of 500 steps with 10 multiple walkers (MW). We display the sampling of all the walkers in Figure S3.

The Funnel-Metadynamics parameters were set to 1.8 nm for the switching point between cone and cylinder, 0.55 radians for the angle of the cone, and 0.1 nm for the radius of the cylinder (zcc, alpha, and rcyl, respectively). We point out that positioning of the funnel potential on the trypsin system was carefully analysed in order to reproduce the same condition of our previous work (Limongelli et al., 2013) and favour a rigorous comparison of the results. The entire FM calculation lasted 800 ns (80 ns per MW) and the procedure is carefully described in the paper of the FMAP protocol (Raniolo and Limongelli, 2020). A comparison of the different behaviours of benzamidine in the binding pocket between the GAFF topology and the *ad hoc* one is offered in figure S2, where it is represented the torsion angle values representing the orientation of benzamidine relative to trypsin sampled in the two simulations.

From these plots it can be seen that at variance with GAFF, in the *ad hoc* simulations benzamidine has a reduced conformational freedom, mostly visiting states close to the absolute free-energy minimum ($\sim$38 vs. $\sim$70 percent of the time in the π/2 to π region, respectively), which represents the ligand binding mode (state A in Figure 5 of the main text). The different behaviour is explained by the diverse ligand dihedral angle parameters with higher energy barriers separating the different ligand conformational states in the *ad hoc* topology.

Further information on the procedure and the analysis of the FM calculations on the benzamidine/trypsin system can be found in our protocol manuscript (Raniolo and Limongelli, 2020).

REFERENCES

Limongelli, V., Bonomi, M., and Parrinello, M. (2013). Funnel metadynamics as accurate binding free-energy method. *Proc. Natl. Acad. Soc. USA* 110, 6358–6363. doi:10.1073/pnas.1303186110

Raniolo, S. and Limongelli, V. (2020). Ligand binding free-energy calculations with funnel metadynamics. *Nat. Protoc.* 15, 2837–2866. doi:10.1038/s41596-020-0342-4

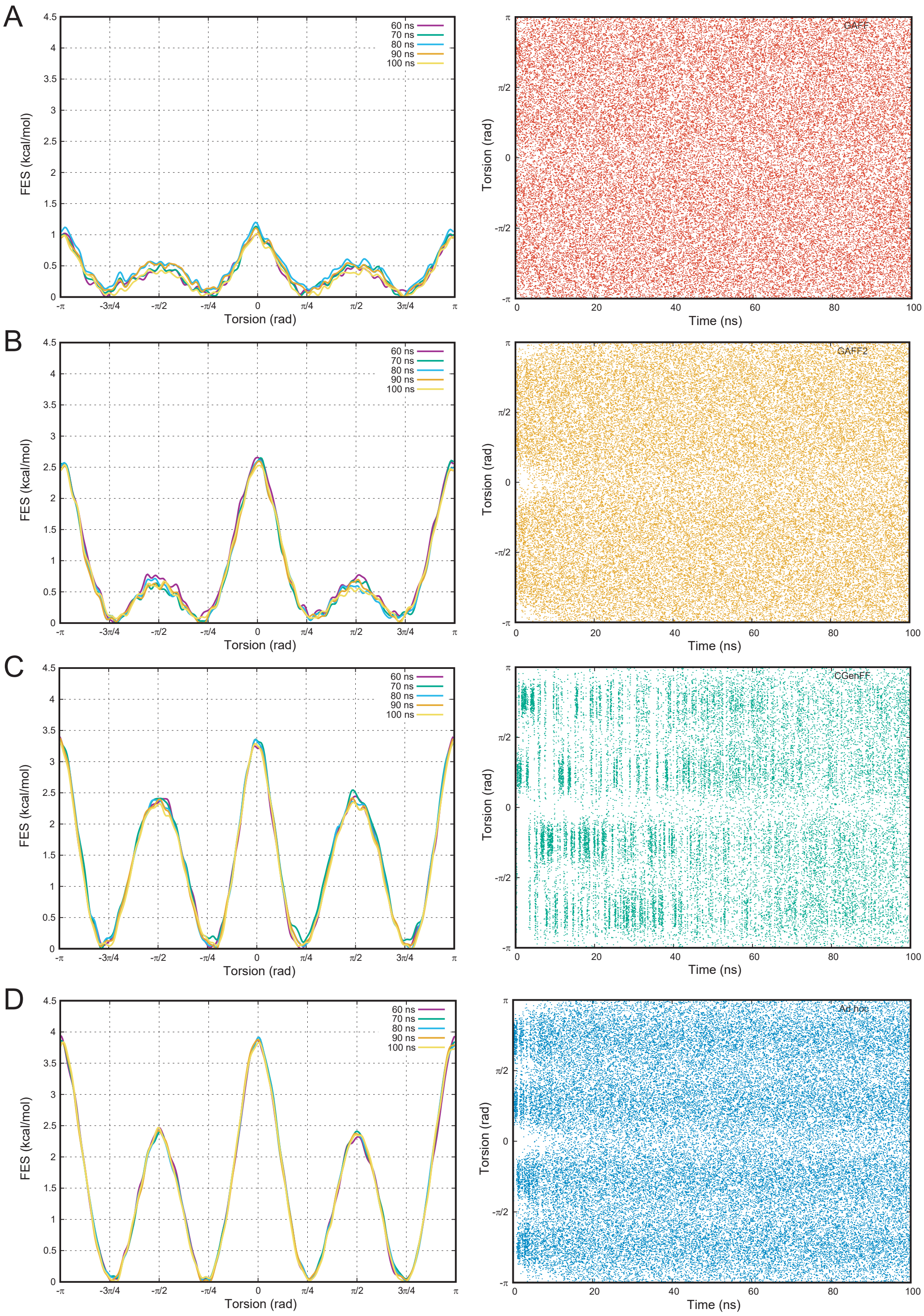

Figure S1. Plots of the free-energy (*Left*) and the torsion CV sampling (*Right*) as a function of the simulation time computed for GAFF (A), GAFF2 (B), CGenFF (C), and the *ad hoc* topology (D).

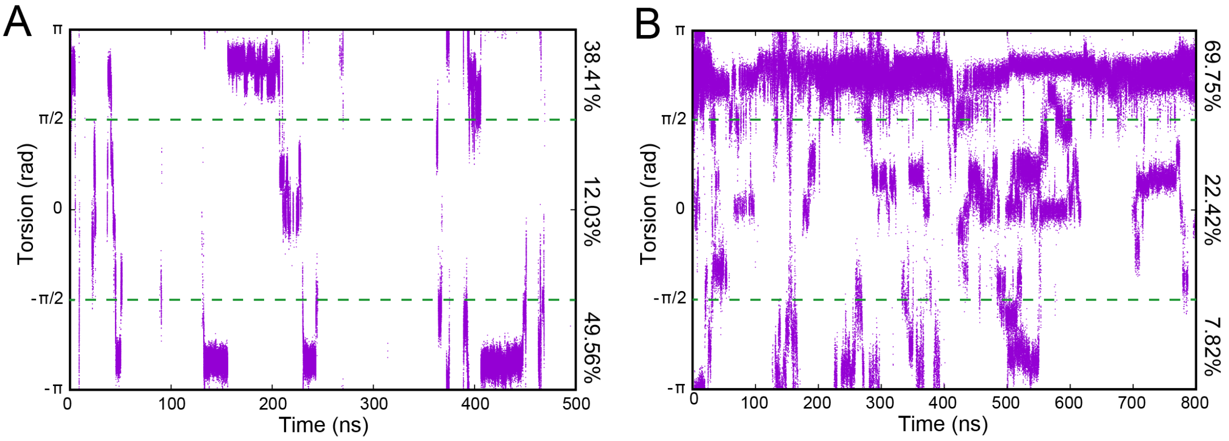

Figure S2. Exploration of the torsion angle CV describing the orientation of benzamidine relative to the trypsin binding site during the FM calculations using the (A) GAFF (Limongelli et al., 2013) and (B) *ad hoc* topology. In order to favour the comparison, only states with the ligand inside the binding pocket (i.e., distance CV lower than 1 nm) were selected, while the green dashed lines indicate the torsion values representing the crystallographic binding pose and the alternative binding mode (from $\pi/2$ to π and from $-\pi$ to $-\pi/2$, respectively). Percentage of the simulation time spent in the different regions of the torsion angle phase space are shown on the right of the plots.

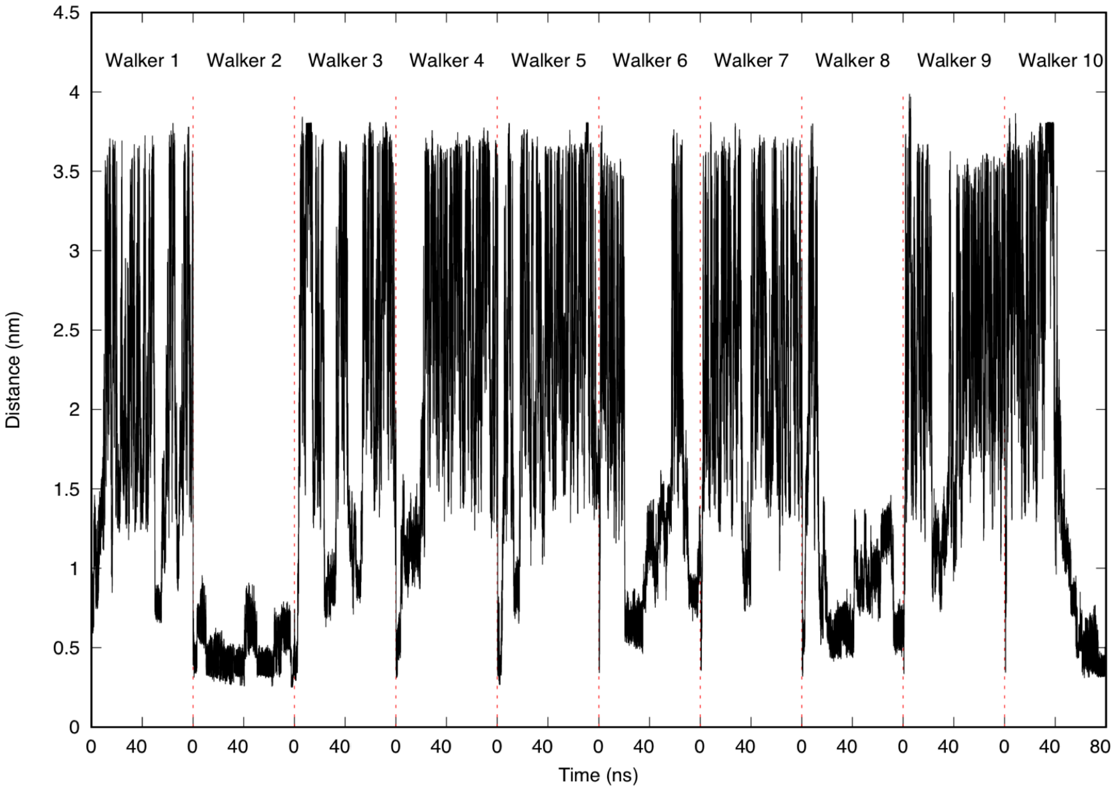

Figure S3. Sampling of the distance CV as a function of the FM simulation time in the case of the benzamidine–trypsin system. The plot is divided by red markers delimiting each of the ten walkers employed. Image taken from Figure 4 of Raniolo and Limongelli (2020) ~~(Raniolo and Limongelli, 2020)~~.
